# Impact of COVID-19 pandemic on business operations of Taiwan High Speed Rail and 7-Eleven stores

**DOI:** 10.1038/s41598-023-34111-0

**Published:** 2023-04-26

**Authors:** Shih-Feng Liu, Hui-Chuan Chang, Yu-Ping Chang, Ho-Chang Kuo, Yuh-Chyn Tsai, Meng-Chih Lin

**Affiliations:** 1 Department of Respiratory Therapy, Kaohsiung Chang Gung Memorial Hospital, Kaohsiung, 833 Taiwan; 2 Division of Pulmonary and Critical Care Medicine, Department of Internal Medicine, Kaohsiung Chang Gung Memorial Hospital, #123, Ta-Pei Road, Niaosong District, Kaohsiung, 833 Taiwan; 3 Medical Department, College of Medicine, Chang Gung University, Taoyuan, 333 Taiwan; 4 Department of Pediatrics, Kaohsiung Chang Gung Memorial Hospital, Kaohsiung, 833 Taiwan

**Keywords:** Environmental social sciences, Medical research

## Abstract

Governmental non-pharmaceutical interventions (NPIs) and concerns regarding COVID-19 infection greatly affected population mobility during the COVID-19 pandemic. This study analyzed the effect of the COVID-19 pandemic on the business operations of Taiwan High Speed Rail (THSR) and 7-Eleven stores in Taiwan. We collected data from COVID-19 Mobility Reports published by Google, the Our World in Data website, and the monthly financial reports of THSR and 7-Eleven stores. The findings revealed that the mean population mobility at transit stations decreased by over 50% during the pandemic. Changes in population mobility were significantly associated with the reproduction rate (7-day rolling average) and with the daily number of new confirmed cases per million people (7-day rolling average). The operating income of THSR was significantly associated with the decrease in population mobility at transit stations. The monthly and annual operating income of THSR in 2020, 2021, and 2022 (during the pandemic) were significantly lower than those in 2019 (before the pandemic). THSR’s monthly operating income was lowest compared with the 2019 value during the Alpha variant period (89.89% lower). No significant correlation was noted between the operating income of 7-Eleven stores and population mobility. Moreover, no significant differences were discovered between the monthly and annual operating incomes of 7-Eleven stores in 2019 and those in 2020, 2021, and 2022. Implementation of the policy of coexistence with the virus by the Taiwanese government began in May 2022, and from May 2022 to October 2022, the monthly income of 7-Eleven stores was higher than that in 2019 whereas the monthly income of THSR began lower than and then slowly increased to the level in 2019. In conclusion, the operating performance of THSR was closely related to population mobility and government NPIs, whereas the operating performance of 7-Eleven stores was less strongly affected by NPIs. These stores increased their operating income by providing e-commerce and delivery services; they thus remained popular in the community.

## Introduction

COVID-19 is a highly transmissible disease caused by the SARS-CoV-2 virus. By May 2022, the number of confirmed COVID-19 cases globally had reached 500 million, and the disease had caused over 6 million deaths^1^. Increased vaccine coverage has led to decreases in the number of severe cases and deaths in patients with COVID-19^2,3^. However, the rapid evolution and mutation of the SARS-CoV-2 virus have resulted in virus variants, each with varying transmissibility and morbidity. The number of confirmed cases caused by spike protein variants has continued to increase in several countries and has even resulted in breakthrough infections^4,5^. This phenomenon indicates that some variants infect vaccinated individuals or those who have developed immunity through natural infection and may even suppress the effects of antibody-based treatments^6^.

The high transmissibility of the SARS-CoV-2 virus has limited the public’s ability to move around freely^7,8^ and thus reduced interactions between both people and nations. During 2019–2021, governments globally made rolling adjustments to COVID-19 pandemic (hereafter referred to as “pandemic”) prevention policies in accordance with the severity of the pandemic. For example, some governments have adopted strict pandemic prevention measures, including shutting down schools and implementing citywide and nationwide lockdowns. However, these measures have caused various societal and economic problems^9–11^. Additionally, increases in the pandemic morbidity rate and individual behavior driven by fear have considerably reduced population mobility at various venues. Labor shortages, traffic disruptions, workplace shutdowns, trade and travel restrictions, and international border closures have considerably affected many economies^12^. These changes have particularly affected physical stores and industries that require consumers to physically visit them. This study analyzed online data on population mobility collected from various venues, on pandemic development, and on the operating performance of Taiwan High Speed Rail (THSR) and 7-Eleven stores in Taiwan during the pandemic to determine the effect of the pandemic on population mobility and the operating performance of various industries.

## Materials and methods

### Study design

This study collected data on the Taiwanese population’s mobility during the pandemic and on the pandemic’s development from COVID-19 Mobility Reports published by Google (https://www.google.com/covid19/mobility/)^13^ and Our World in Data (https://ourworldindata.org/covid-cases)^14^ website. In addition, the monthly financial reports of THSR^15^ and 7-Eleven stores^16^ for the period 2019–2022 were obtained from each company’s website. Data from January 2020 to February 2022 were compiled and analyzed to determine the impact of the pandemic on the business operations of THSR and 7-Eleven stores in Taiwan.

### COVID-19 pandemic trend in Taiwan

With reference to the curve diagrams of daily new confirmed cases per million people (7-day rolling average) published by the Taiwan Centers for Disease Control and the Our World in Data website, the pandemic trend was divided into three periods: the pre-existing variant, Alpha variant, and Omicron variant periods.

### Study population

Data on confirmed COVID-19 cases uploaded by the Taiwan Centers for Disease Control to the Our World in Data website were deidentified to enable the dissemination of comprehensive information on the daily number of new confirmed cases, the reproduction rate, and case fatalities.

### Changes in population mobility

Changes in population mobility were examined using data from COVID-19 Mobility Reports published by Google. Data were collected for the period from January 3, 2020, to February 6, 2020 (5 weeks). The daily mean for each week was used as a benchmark value. The benchmark value was used to evaluate changes in population mobility and the duration of visits to each venue. The changes were computed, and data were deidentified to protect individuals’ privacy.

### Ethics

In accordance with the regulations of the Ministry of Health and Welfare, this study was exempted from ethical review because it was conducted using legal and publicly available information and performed for a publicly known purpose. This study was approved by the Human Experiment Ethics Committee/Institutional Review Board of Chang Gung Memorial Hospital (approval number: 202000966B1).

### Statistical analysis

COVID-19 Community Mobility Reports published by Google provide insights into the trends in movement of various populations at different locations. These data are expressed as the mean, median, and maximal absolute values and percent change over time relative to the baseline value. Data on daily new confirmed cases are presented as mean, median, and maximal values. In this study, Spearman correlation analysis was performed to determine the correlation between population mobility and daily new confirmed cases per million people (7-day rolling average) and that between population mobility and the reproduction rate (7-day rolling average). Statistical analysis was performed using STATA Version 12 (College Station, TX, USA). A two-sided p value of < 0.05 was considered statistically significant.

## Results

### Changes in mean population mobility during each period of the pandemic

**Figure 1:**
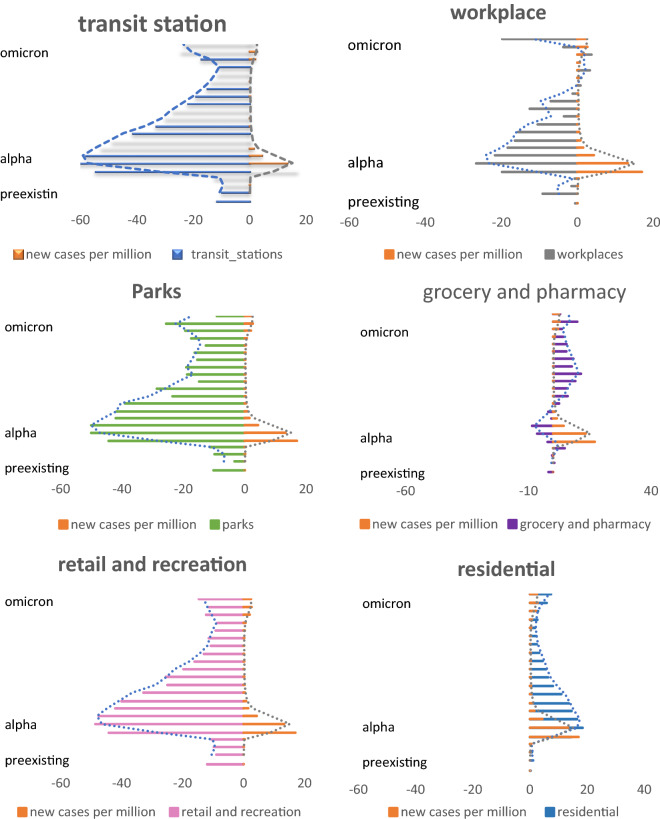
Changes in population mobility at various places during the pre-existing. Alpha, and Omicron variant periods.

Changes in mean population mobility were greatest during the Alpha variant period, followed by the Omicron and pre-existing variant periods (Fig. 1). During the Alpha variant period, the highest decrease in mean population mobility was observed at transit stations (over 50%), followed by parks, workplaces, grocery stores, and pharmacies. The mean population mobility in residential areas slightly increased. During the Omicron variant period, the mean population mobility decreased by approximately 20% at transit stations and parks but increased at grocery stores, pharmacies, and residential areas. During the pre-existing variant period, the highest decrease (approximately 10%) in mean population mobility was noted at transit stations, followed by parks and workplaces. The mean population mobility at grocery stores, pharmacies, and residential areas increased slightly. However, the increases were smaller than 1% (Fig. 1).

### Correlations of mean population mobility with daily new confirmed cases per million people (7-day rolling average) and reproduction rate (7-day rolling average) before and after implementation of the coexistence with the virus policy

The Alpha variant period had the highest (25.1) peak of daily new confirmed cases per million people (7-day rolling average), followed by the Omicron variant (6.39) and pre-existing variant (0.81) periods. A significant correlation was discovered between the daily number of new confirmed cases per million people (7-day rolling average) and population mobility at each type of location (p < 0.001; Table 1).

**Table 1:** Relationships of changes in population mobility at various locations with daily number of new confirmed cases per million people (7-day rolling average) (A) and reproduction rate (7-day rolling average) (B) .

	Coefficient	95% CI	P value
Before co-existence			
(A)			
Retail and recreation	−0.528	−0.583 to −0.472	0.000
Grocery and pharmacy	−0.192	−0.267 to −0.117	0.000
Parks	−0.537	−0.595 to −0.479	0.000
Transit stations	−0.502	−0.556 to −0.447	0.000
Workplaces	−0.181	−0.257 to −0.105	0.000
Residential	0.514	0.456 to 0.568	0.000
(B)			
Retail and recreation	−0.013	−0.069 to 0.094	0.30
Grocery and pharmacy	0.560	0.503 to 0.616	0.000
Park	−0.079	−0.155 to −0.004	0.040
Transit stations	0.266	0.194 to 0.338	0.000
Workplaces	−0.164	−0.087 to 0.241	0.000
Residential	−0.201	−0.277 to 0.124	0.000
After co-existence (2022.5–2022.10)			
(A)			
Retail and recreation	−0.608	−0.723 to −0.492	0.000
Grocery and pharmacy	−0.328	−0.285 to −0.171	0.000
Parks	−0.532	−0.655 to −0.407	0.000
Transit stations	−0.485	−0.632 to −0.339	0.000
Workplaces	−0.146	−0.317 to −0.237	0.092
Residential	0.295	0.128 to 0.461	0.001
(B)			
Retail and recreation	−0.065	−0.242 to −0.111	0.469
Grocery and pharmacy	0.1359	−0.025 to −0.297	0.098
Park	−0.255	−0.412 to −0.099	0.001
Transit stations	0.128	−0.061 to 0.318	0.184
Workplaces	−0.037	−0.209 to 0.136	0.678
Residential	0.067	−0.110 to 0.244	0.458

The Alpha variant period (2.016) had the highest peak reproduction rate (7-day rolling average), followed by the Omicron (1.69) and pre-existing variant (1.29) periods. The reproduction rate (7-day rolling average) was significantly associated with the mean population mobility at transit stations (p < 0.001), grocery stores and pharmacies (p < 0.001), workplaces (p < 0.001), residential areas (p < 0.001), and parks (p = 0.04) but not that at retail stores or recreation venues (p = 0.3; Table 1). For the period after implementation of the Taiwan government’s policy of coexistence with the virus (i.e., from May 2022 to October 2022), a significant correlation was observed between population mobility at various locations and the number of new confirmed cases except for population mobility at workplaces, which was the same as that before the policy. However, the relationship between population mobility at various locations—grocery stores, pharmacies, transit stations, workplaces, and residential areas—and the reproduction rate was significantly different before versus after the policy implementation; the correlation changed from meaningful to nonexistent (Table 1).

### Correlation between THSR operating income and mean population mobility

**Figure 2:**
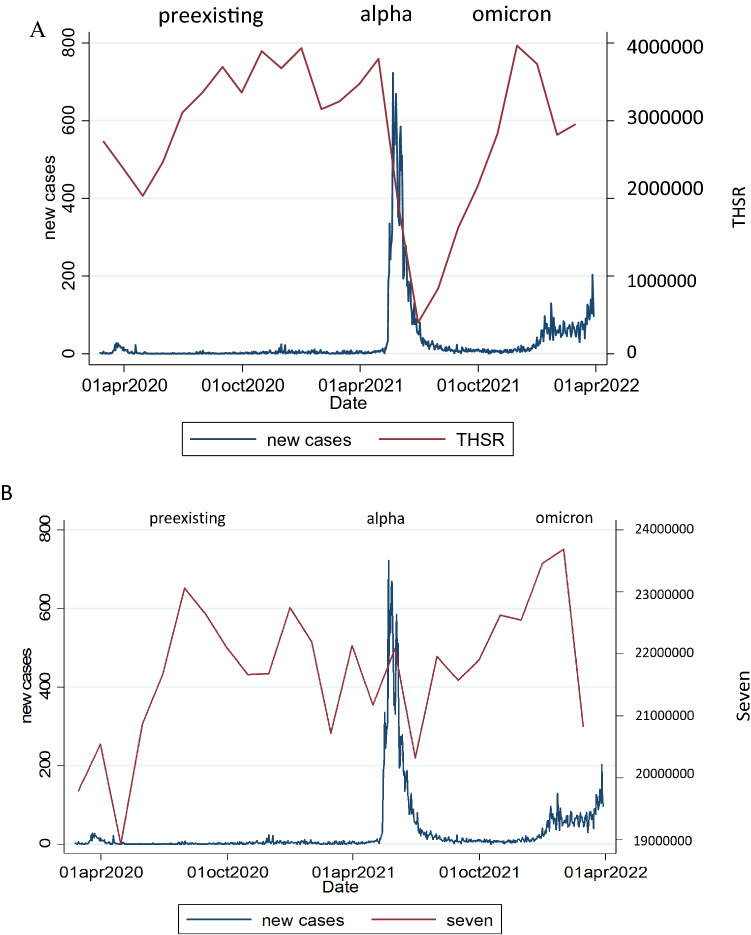
Relationship of daily number of new confirmed COVID-19 cases per million people (7-day rolling average) with the monthly operating income of (**A**) THSR and (**B**) 7-Eleven stores.

Significant correlations were noted between the operating income of THSR and the mean population mobility at transit stations (p < 0.001), grocery stores and pharmacies (p < 0.001), retail stores and recreation venues (p = 0.004), and residential areas (p = 0.049). However, no significant correlations were discovered between the operating income of THRS and the mean population mobility at parks (p = 0.145) and workplaces (p = 0.358; Table 2). The monthly operating income of THSR in 2020, 2021, and 2022 (i.e., during the pandemic) was significantly lower than that in 2019 (i.e., before the pandemic). The highest drop (89.89%) in monthly operating income relative to that in 2019 was observed during the Alpha variant period. The highest drops in the monthly operating income of THSR during the pre-existing variant and Omicron variant periods relative to that in 2019 were 49.83% and 26.46%, respectively (Fig. 2A). For the period beginning after implementation of the Taiwan government’s policy of coexistence with the virus (i.e., from May 2022 to October 2022), the relationship between change in population mobility at transit stations and the monthly operating income of THSR was stronger than that before the implementation of this policy (coefficient = 0.943 > 0.728).

**Table 2:** Relationship of changes in population mobility at various locations with the monthly operating income of THSR.

	Coefficient	95% CI	P value
Before co-existence			
Retail and recreation	0.541	0.172 to 0.911	0.004
Grocery and pharmacy	0.392	0.032 to 0.752	0.033
Park	0.316	−0.109 to 0.741	0.145
Transit stations	0.728	0.476 to 0.980	0.000
Workplaces	0.195	−0.221 to 0.611	0.358
Residential	−0.390	−0.780 to −0.001	0.049
After co-existence (2022.5–2022.10)			
Retail and recreation	0.829	0.203 to 1.453	0.009
Grocery and pharmacy	0.829	0.248 to 1.410	0.005
Park	0.486	−0.489 to 1.460	0.329
Transit stations	0.943	0.593 to 1.292	0.000
Workplaces	0.714	0.024 to 1.404	0.042
Residential	−0.886	−1.347 to −0.445	0.000

### Correlation between the operation income of 7-Eleven stores and mean population mobility

For the period before the beginning of implementation of the Taiwan government’s policy of coexistence with the virus, no significant correlation was found between the operating income of 7-Eleven stores and population mobility (Table 3). After the beginning of this policy’s implementation, the monthly operating income of 7-Eleven stores was significantly related to population mobility at retail stores, recreation venues, grocery stores, pharmacies, parks, and workplaces.

**Table 3:** Relationship of changes in population mobility at various locations with the monthly operating income of 7-Eleven stores.

	Coefficient	95% CI	P value
Retail and recreation	0.067	−0.348 to 0.482	0.752
Grocery and pharmacy	0.397	−0.003 to 0.798	0.052
Park	−0.203	−0.617 to 0.212	0.338
Transit stations	0.083	−0.305 to 0.471	0.674
Workplaces	−0.184	−0.627 to 0.259	0.415
Residential	0.104	−0.338 to 0.547	0.644
After co-existence (2022.5–2022.10)			
Retail and recreation	0.829	0.240 to 1.417	0.006
Grocery and pharmacy	0..829	0.232 to 1.425	0.006
Park	0.943	0.571 to 1.315	0.000
Transit stations	0.714	−0.014 to 1.443	0.055
Workplaces	0.714	0.015 to 1.413	0.045
Residential	−0.429	−1.493 to 0.636	0.430

The monthly operating income of 7-Eleven stores in April 2020 (the pre-existing variant period) was 8.45% lower than that in April 2019. Relative to the monthly operating income of 7-Eleven stores in April 2019, the monthly operating income in the same month during the Alpha variant period was 5.8% lower. However, relative to the overall monthly operating income in 2019, that in 2020 and 2021 was 1.03% and 2.69% higher, respectively (Fig. 2B).

### Comparison of the monthly operating incomes of 7-Eleven stores and THSR from 2019 to 2022

The monthly income of 7-Eleven stores in 2020 was approximately − 8.45% to 8.51% higher than that in 2019, whereas that in 2021 was approximately − 5.8% to 8.89% higher than that in 2019. Compared with the annual income in 2019, the annual income in 2020 and 2021 was 1.03% and 2.69% higher, respectively; however, these increases were not significant (Table 4). The monthly income of THSR in 2020 was approximately − 49.83% to 19.13% higher than that in 2019, whereas that in 2021 was approximately − 89.89% to 0.41% higher than that in 2019. Compared with the annual income in 2019, that in 2020 and 2021 was 17.62% and 34.52% lower, respectively, which were significant differences (Table 5). For the period after implementation of the policy of coexistence with the virus (i.e., from May 2022 to October 2022), the monthly income of 7-Eleven stores was still higher than that in 2019 and that of THSR slowly increased to the level in 2019.

**Table 4:** Monthly operating income of 7-Eleven stores from 2019 to 2022.

	2019	2020	%	2021	%	2022	%
Jan	20,923,655	22,704,519	8.51	22,193,730	6.07	23,694,774	13.24
Feb	19,454,060	19,797,798	1.77	20,724,048	6.53	20,832,985	7.09
Mar	20,707,681	20,547,431	−0.77	22,135,425	6.89	23,208,930	12.08
Apr	20,686,276	18,938,810	−8.45	21,180,179	2.39	23,338,005	12.82
May	21,688,271	20,873,614	−3.76	22,082,804	1.82	23,772,900	9.61
Jun	21,573,376	21,680,106	0.49	20,321,411	−5.80	23,950,195	11.02
Jul	22,385,560	23,060,586	3.02	21,957,391	−1.91	25,537,871	14.08
Aug	22,162,649	22,637,009	2.14	21,578,954	−2.63	25,652,734	15.75
Sep	21,540,458	22,106,331	2.63	21,907,818	1.71	24,656,273	14.46
Oct	21,770,429	21,671,912	−0.45	22,627,169	3.94	24,787,065	13.86
Nov	20,704,519	21,687,275	4.75	22,545,901	8.89		
Dec	22,235,642	22,750,749	2.32	23,462,796	5.52		
Total	255,832,576	258,456,140	1.03	262,717,626	2.69

**Table 5:** Monthly operating income of THSR from 2019 to 2022.

	2019	2020	%	2021	%	2022	%
Jan	3,768,062	4,488,704	19.13	3,148,933	−16.43	2,950,990	−21.68
Feb	4,012,567	2,733,939	−31.87	3,247,104	−19.08	2,817,257	−29.79
Mar	3,981,561	2,377,640	−40.28	3,471,325	−12.81	3,222,095	−19.07
Apr	4,054,767	2,034,083	−49.83	3,798,528	−6.32	2,748,579	−32.21
May	3,841,864	2,468,406	−35.75	1,901,903	−50.50	1,790,496	−53.40
Jun	3,969,896	3,102,809	−21.84	401,290	−89.89	2,126,536	−46.43
Jul	3,922,972	3,364,502	−14.24	840,306	−78.58	2,991,101	−23.75
Aug	3,879,227	3,694,189	−4.77	1,621,143	−58.21	3,366,023	−13.23
Sep	3,876,683	3,364,751	−13.21	2,146,949	−44.62	3,423,080	−11.70
Oct	4,044,502	3,897,049	−3.65	2,832,491	−29.97	3,697,609	−8.58
Nov	3,952,094	3,677,230	−6.95	3,968,202	0.41	3,753,298	−5.03
Dec	4,203,195	3,933,903	−6.41	3,729,713	−11.26		
Total	47,507,390	39,137,205 (P < 0.000)	−17.62	31,107,887 (P < 0.001)	−34.52		

## Discussion

This study revealed that during the pandemic, population mobility was most changed in the Alpha variant period, followed by the Omicron variant and pre-existing variant periods. During the Alpha variant period, population mobility at transit stations decreased by over 50%. The operating income of THSR was positively associated with population mobility at other locations, including transit stations. In addition, the monthly and annual operating incomes of THSR during the Alpha variant period were significantly lower than those in 2019 (i.e., before the pandemic). The greatest difference in monthly operating income over the same observation periods during the Alpha variant period and in 2019 was approximately 90%. However, the operating income of 7-Eleven stores was not significantly associated with population mobility. A comparison of the monthly operating income of 7-Eleven stores in 2019 with those in 2020 and 2021 revealed that the monthly operating income for each observation period in 2020 and 2021 was slightly higher than the equivalent income in 2019. Furthermore, the annual operating incomes of 7-Eleven stores in 2020 and 2021 were higher than that in 2019.

The pandemic has considerably affected population mobility. This effect is attributable to the transmissibility and morbidity of the SARS-CoV2 virus, the national vaccination coverage rate, the availability of antiviral drugs and COVID-19 rapid test kits, and the implementation of pandemic prevention policies by the government. For example, because vaccination coverage for COVID-9 is high, the Taiwanese government replaced its “zero-tolerance” strategy with a “coexist with the virus” strategy after the rate of severe cases decreased. Therefore, the government relaxed various pandemic prevention measures. However, the consistent mutation of the SARS-CoV-2 virus has resulted in the emergence of new Omicron variants in Europe and the United States. The BA.4 and BA.5 variants may replace the BA.1 and BA.2 variants as the dominant strains^17–19^. Whether these new variants will cause another wave of severe cases, increase the morbidity rate, or cause a breakthrough number of infections should be examined in future research.

The pandemic exerted different effects on the business operations of THSR and 7-Eleven stores. This is because THSR requires consumers to personally interact with its services. By contrast, although some of 7-Eleven’s services are provided in physical stores, 7-Eleven offers flexible services that can suit consumer demands. For example, during the pandemic, 7-Eleven promoted services such as take-out delivery, e-commerce, and provision of multifunctional private rooms. Additionally, because most streets in Taiwan have 7-Eleven stores, the government utilized the convenience of 7-Eleven stores to implement pandemic prevention activities, including its name-based mask rationing plan and selling of rapid test kits and pandemic prevention equipment. Moreover, 7-Eleven stores expanded their services related to deliveries and selling commodities, collaborated with banks to promote cashless payment services, and implemented suitable adaptations to the pandemic as it developed. However, THSR failed to effectively and flexibly implement measures to adapt to the pandemic. When Taiwan was affected by the pandemic, Taiwanese citizens did not want to violate pandemic policies and travel because of their generally law-abiding mindset. This resulted in THSR losing income and forced the company to reduce its expenditure. In this study, the two companies were used as examples to discuss changes in company marketing policies during the pandemic. Pandemic-induced changes in population mobility considerably increased the business performance of companies in the e-commerce, online gaming, and virtual reality fields. However, these changes led to a considerable reduction in the business performance of companies in the tourism, hotel, and catering industries.

The impact of nonpharmaceutical interventions (NPIs) implemented by the government is a crucial factor^20,21^. Before enactment of the coexistence with the virus policy, Taiwan implemented several lockdown policies, such as stay-at-home orders and travel bans, which may have affected the revenue of 7-Eleven stores and THSR. The revenue of both 7-Eleven stores and THSR increased after introduction of the virus coexistence policy, even the Omicron variant was prevalent in Taiwan from May 2022 to October 2022. This indicates the importance of NPIs and the spirit of the people of Taiwan to abide by the law. However, by contrast, the performance of 7-Eleven stores was higher before the implementation of the coexistence with the virus policy than it was in 2019, indicating that 7-Eleven stores made satisfactory adjustments to their operations, such as the expansion of e-commerce activities. Taiwan’s 7-Eleven convenience stores remained popular in the community.

This study has several limitations. First, this study divided the pandemic into three periods. These divisions were made on the basis of the curve diagrams of daily new confirmed cases per million people (7-day rolling average). Therefore, the findings of this study do not reflect small-scale changes. Furthermore, only data for up to February 1, 2022, were downloaded from the Our World in Data website. Because the Taiwanese government shifted its pandemic prevention policy from a zero-tolerance strategy to a coexistence with the virus strategy after the emergence of new confirmed cases of the Omicron variant, this study did not included data from after February 1, 2022. Second, data on population mobility were obtained from COVID-19 Mobility Reports published by Google. Because some people do not use Google software on their mobile phone or occasionally turn off their mobile phone, the collected data may not completely represent the mobility of all Taiwanese residents. Third, minor inconsistencies may have existed between the pandemic statistics provided by the Our World in Data website and the actual statistics. However, it is unlikely that this would have affected the overall accuracy of our data.

## Conclusion

The pandemic affected population mobility at various types of location in Taiwan, which in turn influenced the business operations of many companies. In this study, THSR and 7-Eleven stores were used as examples for analysis. The findings revealed that the business performance of THSR was closely related to population mobility and the impact of NPIs implemented by the government. By contrast, the business performance of 7-Eleven stores was less strongly affected by the NPIs. These stores increased their operating income by offering e-commerce and delivery services; they thus remained popular in the community.

## Data Availability

The data generated and analyzed in this study are included in this article. The data were obtained from the Google online COVID-19 community mobility report (https://www.google.com/covid19/mobility/) and our world in data (ourworldindata.org/coronavirus) THSR (https://en.thsrc.com.tw/corp/e12c0774-d30f-49bd-8541-f1d70a6a161f), and 7-Eleven (https://www.7-11.com.tw/company/ir_en/) financial reports. The datasets used and/or analysed during the current study are available from the corresponding author on reasonable request.

## References

[1] WHO Coronavirus (COVID-19) Dashboard. https://covid19.who.int/ (2022).

[2] Suthar AB (2022). Public health impact of covid-19 vaccines in the US: observational study. BMJ.

[3] COVID-19 Incidence and Death Rates Among Unvaccinated and Fully Vaccinated Adults with and Without Booster Doses During Periods of Delta and Omicron Variant Emergence—25 U.S. Jurisdictions, April 4–December 25, 2021 Weekly/January 28, 2022/71(4), 132–138 (2021). 10.15585/mmwr.mm7104e2 PMC9351531 35085223

[4] UC Davis Health. *Omicron Variant: What We Know About This COVID-19 Strain*. https://health.ucdavis.edu/coronavirus/covid-19-information/omicron-variant (2022).

[5] Bergwerk M (2021). Covid-19 breakthrough infections in vaccinated health care workers. N. Engl. J. Med..

[6] Khandia R (2022). Emergence of SARS-CoV-2 Omicron (B.1.1.529) variant, salient features, high global health concerns and strategies to counter it amid ongoing COVID-19 pandemic. Environ. Res..

[7] Okamoto S (2022). State of emergency and human mobility during the COVID-19 pandemic in Japan. J. Transp. Health.

[8] Zhou Y (2020). Effects of human mobility restrictions on the spread of COVID-19 in Shenzhen, China: A modelling study using mobile phone data. Lancet Digit Health..

[9] Bonaccorsi G (2020). Economic and social consequences of human mobility restrictions under COVID-19. Proc. Natl. Acad. Sci. USA.

[10] Zachreson C (2021). Mapping home internet activity during COVID-19 lockdown to identify occupation related inequalities. Sci. Rep..

[11] Wang J, Kaza N, McDonald NC, Khanal K (2022). Socio-economic disparities in activity-travel behavior adaptation during the COVID-19 pandemic in North Carolina. Transp. Policy (Oxf)..

[12] Shang YF, Li H, Zhang R (2021). Effects of pandemic outbreak on economies: Evidence from business history context. Front. Public Health.

[13] COVID-19 Community Mobility Reports. *See How Your Community is Moving Around Differently Due to COVID-19*. https://www.google.com/covid19/mobility/.

[14] Our World in Data. *Explore the Global Data on Confirmed COVID-19 Cases in Taiwan*. https://ourworldindata.org/covid-cases (2022).

[15] Financial Reports. https://en.thsrc.com.tw/corp/e12c0774-d30f-49bd-8541-f1d70a6a161f (2022).

[16] Annual Reports. https://www.7-11.com.tw/company/ir_en/ (2022).

[17] Callaway E (2022). New Omicron relatives BA.4 and BA.5 offer hints about the future of SARS-CoV-2. Nature.

[18] Mohapatra RK (2022). The recently emerged BA.4 and BA.5 lineages of Omicron and their global health concerns amid the ongoing wave of COVID-19 pandemic—Correspondence. Int. J. Surg..

[19] Yamasoba D (2022). Sensitivity of novel SARS-CoV-2 Omicron subvariants, BA.2.11, BA.2.12.1, BA.4 and BA.5 to therapeutic monoclonal antibodies. bioRxiv.

[20] Sophia, C. *et al*. Tracking the economic impact of COVID-19 and mitigation policies in Europe and the United States. in *IMF Working Papers 2020.125* (2020).

[21] Maloney, W.F. *et al*. Determinants of social distancing and economic activity during COVID-19: A global view. in *World Bank Policy Research Working Paper 9242* (2020).

